# Identifying tinnitus in mice by tracking the motion of body markers in response to an acoustic startle

**DOI:** 10.3389/fnins.2024.1452450

**Published:** 2024-08-07

**Authors:** Mark N. Wallace, Joel I. Berger, Adam Hockley, Christian J. Sumner, Michael A. Akeroyd, Alan R. Palmer, Peter A. McNaughton

**Affiliations:** ^1^Hearing Sciences, School of Medicine, University of Nottingham, Nottingham, United Kingdom; ^2^Human Brain Research Laboratory, Department of Neurosurgery, University of Iowa Hospitals and Clinics, Iowa City, IA, United States; ^3^Cognitive and Auditory Neuroscience Laboratory, Institute of Neuroscience of Castilla y León, University of Salamanca, Salamanca, Spain; ^4^NTU Psychology, Nottingham Trent University, Nottingham, United Kingdom; ^5^Wolfson Sensory, Pain and Regeneration Centre, King’s College London, London, United Kingdom

**Keywords:** gap-induced prepulse inhibition, rodent, guinea pig, Preyer reflex, sodium salicylate

## Abstract

Rodent models of tinnitus are commonly used to study its mechanisms and potential treatments. Tinnitus can be identified by changes in the gap-induced prepulse inhibition of the acoustic startle (GPIAS), most commonly by using pressure detectors to measure the whole-body startle (WBS). Unfortunately, the WBS habituates quickly, the measuring system can introduce mechanical oscillations and the response shows considerable variability. We have instead used a motion tracking system to measure the localized motion of small reflective markers in response to an acoustic startle reflex in guinea pigs and mice. For guinea pigs, the pinna had the largest responses both in terms of displacement between pairs of markers and in terms of the speed of the reflex movement. Smaller, but still reliable responses were observed with markers on the thorax, abdomen and back. The peak speed of the pinna reflex was the most sensitive measure for calculating GPIAS in the guinea pig. Recording the pinna reflex in mice proved impractical due to removal of the markers during grooming. However, recordings from their back and tail allowed us to measure the peak speed and the twitch amplitude (area under curve) of reflex responses and both analysis methods showed robust GPIAS. When mice were administered high doses of sodium salicylate, which induces tinnitus in humans, there was a significant reduction in GPIAS, consistent with the presence of tinnitus. Thus, measurement of the peak speed or twitch amplitude of pinna, back and tail markers provides a reliable assessment of tinnitus in rodents.

## 1 Introduction

There are 26 million people who experience tinnitus in Europe alone, with a significantly higher percentage among countries with greater levels of poverty (Biswas et al., 2022). The most commonly desired treatment modality is an effective drug, with 62% of patients stating that they would be prepared to try medication (Tyler, 2012). Despite this, none of the clinical trials to date have led to a drug that is universally effective (Barth et al., 2021), partly because of the lack of an objective biomarker for tinnitus that can be used in both animal and human studies (McFerran et al., 2019). We have developed a test in guinea pigs based on using the pinna reflex to assess the gap-induced prepulse inhibition of the acoustic startle (GPIAS) and we showed a significant reduction in GPIAS following the induction of tinnitus with sodium salicylate (Berger et al., 2013) or noise exposure (Coomber et al., 2014; Hockley et al., 2020). Humans also show a pinna reflex, as there is a vestigial posterior auricular muscle reflex (Hackley, 2015) which can be measured as a small scalp potential and demonstrates GPIAS (Wilson et al., 2019) despite the pinna being immobile. However, this reflex was not found to be reliable enough for detecting tinnitus in humans (Wilson et al., 2020) and in this study we sought an alternative way of measuring GPIAS that might be suitable for both rodents and humans. Recent evidence has indicated that people with tinnitus show reduced microfacial movements in response to acoustic stimuli when measured with a video camera at a sampling frequency of 120 Hz (Smith et al., 2024). This suggests that motion tracking muscle twitches in 3D, with small reflective markers applied to the face, may also be a useful way of identifying tinnitus in humans.

**Figure 1:**
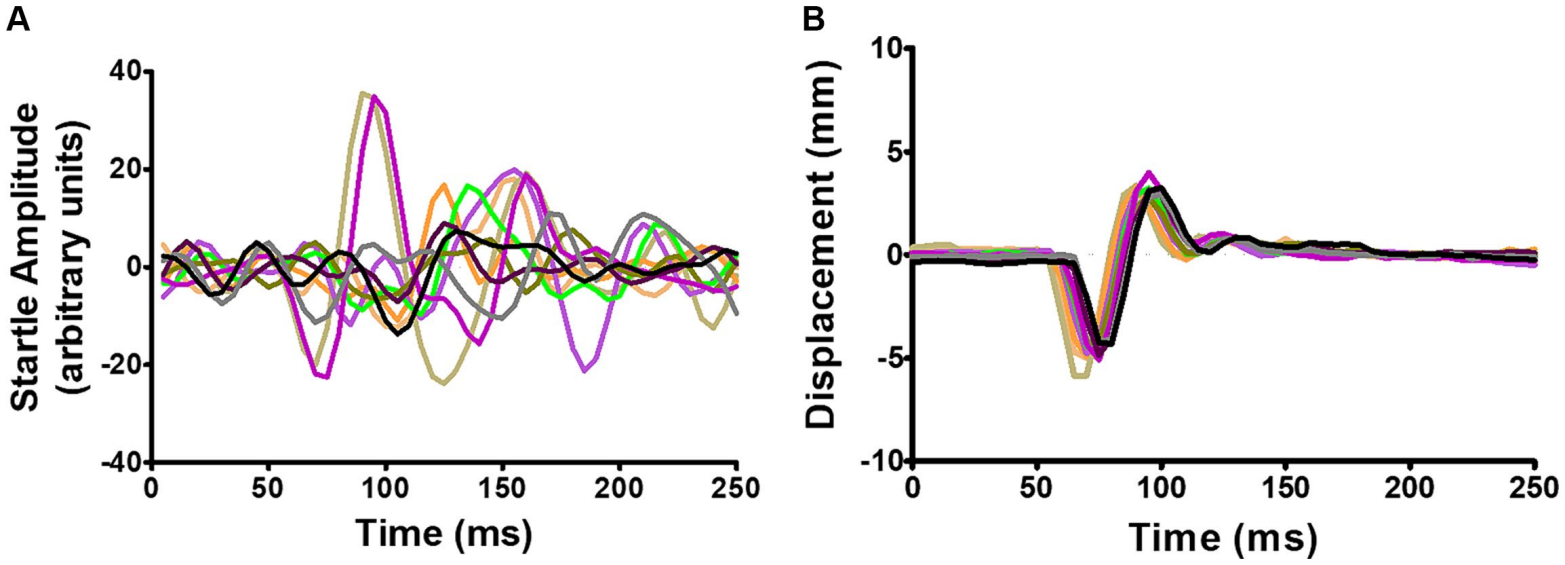
**(A)** Example of recordings from a single-point aluminium load cell showing WBS in response to 10 repeats of a startle pulse at 95 dB SPL. **(B)** Simultaneous recordings of the relative displacement of two ear markers using the Vicon motion tracking system. The Vicon system showed the pinnae movement had a simpler waveform with much less variability. Adapted from Berger et al. (2013).

In small rodents GPIAS is usually detected as a whole-body startle (WBS), while in humans (Fournier and Hébert, 2016) and monkeys (Rogenmoser et al., 2022) it is usually detected as the eyeblink reflex by electromyographic (EMG) recording. EMG recordings are not suitable for freely moving rodents and the acoustic startle is usually measured by placing the animal on a platform for detecting pressure changes, either involving a load cell (Virag et al., 2021), or piezoelectric sensors (Dulawa et al., 1997; Longenecker and Galazyuk, 2012; Schilling et al., 2017; Ioannidou et al., 2018). However, the response magnitude of the WBS habituates rapidly and after ~5 repeats diminishes to a level that is difficult to distinguish from background movements (Figure 1A). A waveform template (Grimsley et al., 2015) or more sophisticated methods of waveform analysis involving machine learning algorithms (Fawcett et al., 2020, 2023) have only had partial success.

In the guinea pig we have successfully measured the acoustic startle reflex using a motion tracking system (Vicon Motion Systems, Oxford, United Kingdom) to track small reflective markers placed on the pinna, which has allowed us to measure the pinna reflex directly as a non-habituating component of the acoustic startle response with a much greater signal-to-noise ratio than the whole-body startle (Figure 1B; Berger et al., 2013). Inexpensive motion tracking systems (e.g., OptiTrack Flex 3; Natural Point, Corvallis, OR, United States) have more recently become available, in part due to software developments linked to the video gaming industry (Guess et al., 2022). The development of mouse genetic knockouts may identify genes relevant for tinnitus (Yu et al., 2016), but unlike the guinea pig, mice have very sensitive pinnae and object to having markers placed on them. Thus, the pinna reflex does not seem suitable for measuring GPIAS in either mice or humans and a more suitable component of the startle reflex was sought that would be suitable for mice genetic models.

The startle reflex simultaneously produces twitches in multiple groups of muscles throughout the face, neck, torso and limbs and this produces a wide variety of movements in both humans (Wilkins et al., 1986; Smith et al., 2024) and mice (Pantoni et al., 2020; Clayton et al., 2024). In this study we placed reflective markers on freely moving guinea pigs and mice in various positions, to test which locations are most suitable for detecting the movements forming the startle reflex and which could be used for measuring GPIAS. In guinea pigs the lateral edge of the pinna was confirmed as a reliable location for recording GPIAS. Mice generally did not tolerate reflective markers placed on the head or pinna but recordings from their back and tail gave robust GPIAS which was reduced when tinnitus was induced with sodium salicylate.

## 2 Materials and methods

### 2.1 Animals

Male Dunkin-Hartley guinea pigs (*n* = 18) were obtained from an approved supplier (Marshall BioResources) at a weight of 300–350 g and all measurements were completed before they were 6 months old. In previous studies, where we used reflective markers to study GPIAS using the Preyer reflex, we had always used mixed male and female groups and never noticed any differences in the startle response linked to sex (Berger et al., 2013; Coomber et al., 2014; Hockley et al., 2020). The current guinea pigs were subsequently noise-exposed and used in a separate drug study where we wanted to avoid variations in sex hormones such as oestrogen that might interfere with the drug response. Thus, only male guinea pigs were used. For the sake of a consistent comparison and to reduce the number of animals used in this study only male mice were used. C57BL/6J mice (strain code 632; *n* = 22) were obtained from Charles River at the age of 3 months. All data was obtained before the mice were 5 months old to minimise the risk of interference from elevated thresholds in this strain which has early onset hearing loss (Spongr et al., 1997). Animals were group-housed in cages with 2–4 individuals on a 12:12 h light:dark cycle with food and water freely available, with temperature maintained in the range of 19–21°C and humidity at 40–70%. All procedures were carried out under authority of the UK Home Office under the Animals (Scientific Procedures) Act 1986. Experiments were run in accordance with the European Communities Council Directive 1986 (86/609/EEC) and the approval of the Animal Welfare and Ethical Review Body at the University of Nottingham, UK.

### 2.2 Acoustic stimulation

Sound files with narrow or broadband background noise and variable amplitudes for the startle pulses were generated as standard 16-bit digital waveform files (.wav) using Adobe Audition (Adobe Systems Incorporated, San Jose, CA). Each file contained a series of 10 gap and 10 non-gap startle pulses in a standard but pseudorandom order at 6 s intervals with a continuous background of broadband noise (BBN) or narrow band noise (4–6 or 16–18 kHz). In our first studies of GPIAS in the guinea pig we followed standard practice and used variable inter stimulus intervals of either 15 or 24 s to minimise habituation (Berger et al., 2013). Subsequently we reduced the interval to a constant 6 s as the responses remained at an acceptable level with only a small amount of habituation. Consequently, the duration of a typical session was reduced from 60 to 20 min. This was a desirable refinement because the animals were stressed by being isolated in a chamber and presented with a series of stimuli designed to startle them and we wanted to keep the duration of the stressful sessions to a minimum. Gaps were 50 ms long (2 ms rise/fall time) and ended 50 ms before the 20 ms long (1 ms rise/fall times) startle pulses composed of BBN (50 Hz to 20 kHz). The soundcard output was taken via a Tascam US-144 interface (TEAC Professional Division, United States) to an Onkyo sound amplifier and presented through a single 25 mm loudspeaker (Tymphany Peerless Gold XT25BG60–04 tweeter with a flat output (±3 dB) up to 40 kHz). Sound pressure level calibration in the centre of the animal cage/bowl was performed using a ½ inch free-field microphone (Bruel & Kjaer Model 4176, prepolarized) connected via a Bruel & Kjaer (B & K) preamplifier to a B & K 2636 measuring amplifier. The amplifier was adjusted until the output corresponded to that of a B & K 4231 sound source which puts out a 94 dB SPL signal. The measured sound level at the walls of the mouse bowl were 5 dB lower than in the centre and there were variations of up to 5 dB over the extent of the guinea pig cage. The fact that none of the animals were restrained meant that the sound levels at the ears could vary by as much as 5 dB within one session. Some of the guinea pigs would sit almost motionless for 30 min while testing was undertaken but others seemed more agitated and moved around for much of the session and this was also generally true of the mice.

### 2.3 Measuring motion

We used a motion-tracking camera system consisting of three infrared OptiTrack Flex 3 cameras (Natural Point, Corvallis, OR, United States), to monitor markers placed on the body. These cameras were placed at 750 mm above the animal in an equilateral triangular pattern, with the cameras 300 mm apart and inside a small acoustic booth with foam wedges on walls and ceiling to reduce reflected sounds (Longenecker and Galazyuk, 2012).

In the mouse setup the loudspeaker was directly above the mouse at a height of 840 mm and the mouse was in a polyethylene bowl with a diameter of 210 mm at the top and a height of 140 mm. This contained a 10 mm layer of sawdust and scattered grains of food such as buckwheat or golden flax, but no water and was similar to the mouse home cage to minimise stress. Mice were unable to climb up the smooth walls and never tried to escape. The guinea pig set up used the same cameras but had an open-topped wire cage with non-reflective paint (310 × 155 × 155 mm). Even large guinea pigs could turn around in the cage. The loudspeaker was at one end of the cage and slightly above it.

Prior to each use, the camera system was optimised using a stuffed dummy with reflective markers to optimise the LED levels and the filter settings to reduce the background signal. Software filters were set to only accept a signal from a small round source. The system was calibrated at least once a week to ensure optimal performance and a triangulation residual mean error of 0.1 mm or less. Reflective markers (3 or 4 mm diameter, MCP1130, NaturalPoint.com) were attached to the fur using Loctite all-purpose adhesive that is acetone/methyl acetate based and easily removed. In the guinea pigs these were attached to a fold of skin at the dorsal edge of the pinna or on the back or the sides of the abdomen or thorax. The guinea pigs sat quietly while the markers were applied without any restraint and apart from occasionally shaking their heads, they never tried to remove a marker during a recording session. By contrast, the mice were generally unwilling to allow markers to be placed on their pinnae without restraint and they usually removed the markers immediately after being released from restraint by shaking their head or scratching with their forepaws. We avoided restraining any of our animals during marker placement as it led to increased stress that could interfere with the GPIAS test. Markers placed on the thorax or abdomen of mice were also usually removed within a few minutes by scratching with the hind paw. The only sites where mice appeared to tolerate the placement of markers was the back and or tail. Thus, in the mice two markers were usually attached to the tail and one to the back or neck but even then, some mice would scratch them off. Other positions were also tried if these sites became sensitized by repeated placement. Repeated placement of markers appeared to be stressful and if no markers were still in place after 10 min the mouse was returned to its home cage. In two unusually compliant mice we did manage to attach smaller (3 mm) markers to both pinnae while they were waking up from a period of sleep. To minimise stress mice were transferred from their cage by either cupped hands or in a small cardboard tube. During marker placement the tails were gently held aloft while the mice held onto the grill on the top of the cage.

Marker positions were recorded at a sampling rate of 100 Hz using OptiTrack Motive 2 software. This rate was adequate to form an accurate measure of the rapid startle response in mice as these usually lasted for over 100 ms. The motion-tracking system triangulated the absolute position of the markers and produced three columns of data for each marker corresponding to the x, y and z coordinates. The data were exported as .csv files and analysed in an Excel spreadsheet or in Matlab^®^ (R2009b, MathWorks, MA, United States).

### 2.4 Measuring the startle response and GPIAS

**Figure 2:**
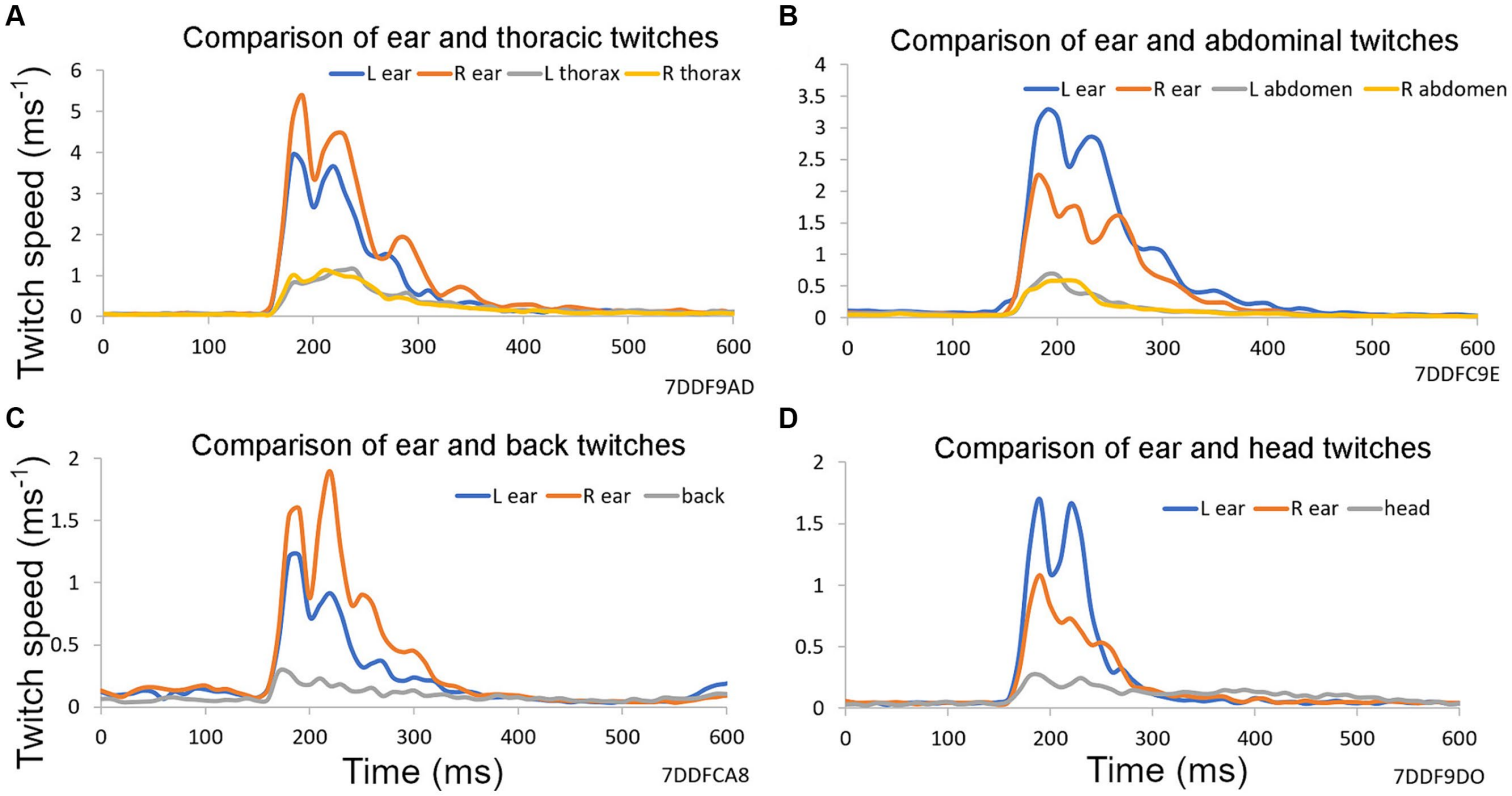
Examples of reflex twitches recorded simultaneously from different parts of the guinea pig body in response to a startle pulse at 90 dB SPL on a background of narrowband noise containing frequencies of 4–6 kHz at 60 dB SPL. These panels show the results from a single trial based on 10 repeats of a startle pulse, without any preceding gap, collected over a period of 2 min. Vertical axis shows first differential of each marker’s position (speed). **(A)** Markers on ears and both sides of the thorax. **(B)** Markers on ears and on both sides of the abdomen. **(C)** Markers on the ears and on the back above the spine. **(D)** Markers on ears and at a central point between them on the head.

Measuring the motion of small reflective markers during the startle reflex was studied as a function of time, but they were analysed in three different ways depending on whether there was a pair of markers available or just one. For two markers placed on paired body parts such as the pinna or abdominal wall the Euclidian distance between them was measured. The change of distance between pinnae was usually biphasic with a negative and positive wave and the displacement measured was the difference between the peaks of the positive and negative waves (see Figure 1B). This measurement worked well in the guinea pig. However, most mice did not permit pairs of markers to be placed on the pinnae or abdomen and in these cases the startle-induced twitches had to be measured with a single marker on a different part of the body. We did this initially in the guinea pig so that we could compare the body twitches with the Preyer reflex (Figure 2). For individual markers the startle was detected by taking the first differential of absolute position (speed). This was plotted from the time of the startle pulse for 150 ms in mice and 250 ms in guinea pigs and analysed in two ways - either as the peak speed of a single marker during the sampling window or by calculating the total movement of a marker during the response. The total movement was calculated by integrating the area under the curve of plotted motion and subtracting a value for spontaneous movement based on the values for an equivalent period immediately prior to the start of the startle twitch. As speed is a scalar value it was not affected by the position or orientation of the animal within the chamber.

GPIAS stimuli were used throughout this study with gaps pseudo randomly presented before 10 out of the 20 startles in a trial. The mean values for the 10 gap and 10 no gap conditions were calculated for each trial and GPIAS calculated as a percentage change by subtracting the mean value for the gap periods from the mean value for the no-gap periods and dividing this by the value for the no gap periods. Each trial took about 2 min and between 3 and 10 trials were repeated in a single session. We found from experience that we could usually obtain statistically significant GPIAS from the equivalent of 5 complete trials with two markers and that was what we aimed for. If four or more markers stayed in place for three trials then we stopped collecting data for that mouse, especially if it seemed a bit stressed and was moving around more than usual. Alternatively, if a mouse had scratched off all but one of its markers or if the responses were small or intermittent then we continued testing for 10 trials. In all cases the product of the number of markers times the number of trials was at least 10. Occasionally a mouse would not cooperate and became very agitated before more than two trials could be collected. In this case it was returned to its cage and a different session completed at a different date. We never combined data from different sessions.

Sessions were always less than 40 min and never repeated on consecutive days on any one animal. The mean results for each trial were combined and analysed with a two-way ANOVA (GraphPad Prism software, version 10) with a significance level of 0.05%. When results were combined from multiple animals to produce growth curves over a range of pulse levels then the mean values for each gap and no-gap condition in individual animals were used to compare the curves with a paired *t*-test.

### 2.5 Administration of sodium salicylate to mice

Administration of sodium salicylate (Sigma, S3007) at high concentrations is known to reliably produce tinnitus in humans and rodents and is a very useful experimental model (Cazals, 2000; Stolzberg et al., 2012). However, it also causes the breakdown of connective tissue and is associated with gastric ulceration and haemorrhage as well as nephrotoxicity when used repeatedly (MacPherson, 2000; Mathews, 2000). For this reason, we administered a single, near-isotonic dose by subcutaneous injection (20 mg/mL in distilled water, 125 mM), at between 200 and 300 mg/kg. Doses above 300 mg/kg were not used in the mouse as they are associated with distress and sometimes death (Yanagawa et al., 2017). Behavioural measurements of GPIAS were made between 2 and 5 h after administration of salicylate when the intracochlear levels of salicylate are near their peak (Cazals, 2000). All mice were killed within 5 h of administering salicylate to avoid any long-term side-effects.

## 3 Results

### 3.1 Comparison of reflex movements from the pinna, thorax, abdomen, back and head in guinea pigs

The acoustic startle response involves many muscles throughout the face, body and limbs. We have previously shown that motion tracking of reflective markers can be used to measure the guinea pig external ear (pinna) reflex as a displacement between the two pinnae (Berger et al., 2013). In Figure 2 the speed of the startle movements (first derivative of position of a single marker) are compared in the left and right ears with the thorax, abdomen, back and head. The pinna is a much more lightweight and mobile structure than the other locations tested, and the pinna reflex had a higher speed than other locations. Movements of the thorax and abdomen were very similar for the two sides, but there was considerable asymmetry between the pinna movements. All positions showed reflex movements with two or more peaks indicating that the response at each site was more complicated than a single muscle twitch.

### 3.2 Comparison of threshold and growth curves for the startle response at different locations in guinea pigs

**Figure 3:**
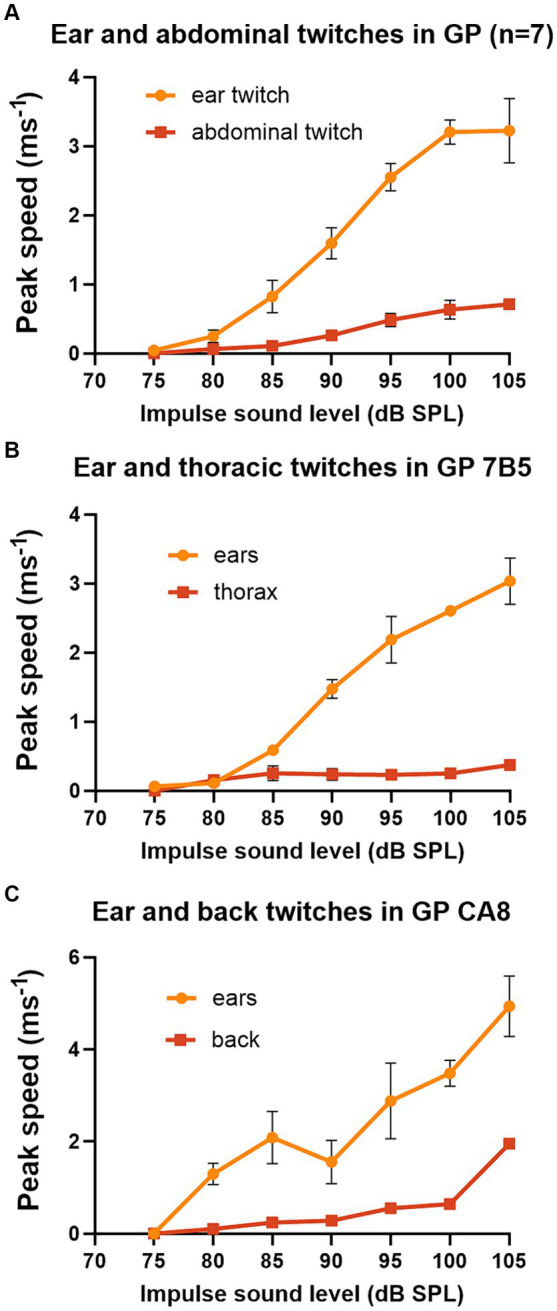
Graphs of peak speed of the twitch with increasing sound level of the startle pulse when markers are placed on different parts of the guinea pig. The startle pulse was superimposed on a background noise (4–6 kHz) at 70 dB SPL. **(A)** There is a sigmoid relationship between sound level and peak speed when the results are combined from both ears and both abdominal markers for a group of 7 guinea pigs. **(B)** A much shallower growth curve was found for markers placed on both sides of the thorax than for markers on the ears in guinea pig 7DDF7B5. **(C)** A comparison of the growth curve for a marker placed on the back above the spine. This measure was initially much shallower than that for the ears but became quite steep between the two highest sound levels tested for guinea pig 7DDFCA8. Error bars show the standard error of the mean (SEM).

The pinna reflex in guinea pigs is usually measured between 90 and 105 dB SPL. Thus, we wanted to measure the threshold and growth curves for the startle responses at different body locations to determine if they were different. Seven of the first group of guinea pigs provided data for a complete series of startle pulses at 5 dB intervals from 75 to 105 dB SPL, where peak speeds were combined for the two ears and the two sides of the abdomen (Figure 3A). Despite the differences in absolute speeds, the curves for the ears and abdomen were fairly similar with thresholds of 75–80 dB SPL and a sigmoid shaped slope which started to flatten out at about 100 dB SPL. The growth curves for the thorax and back twitches were measured in a single animal each and again had similar thresholds of 75–80 dB and increased in speed up to the highest value measured which was at 105 dB SPL (Figures 3B,C). This experiment shows that the twitches of guinea pig abdomen, thorax and back are smaller than the ear, but that the form of the curve of speed vs. sound level of the startle pulse is overall similar.

### 3.3 Comparison of different GPIAS analysis methods for the pinna reflex in guinea pigs

**Figure 4:**
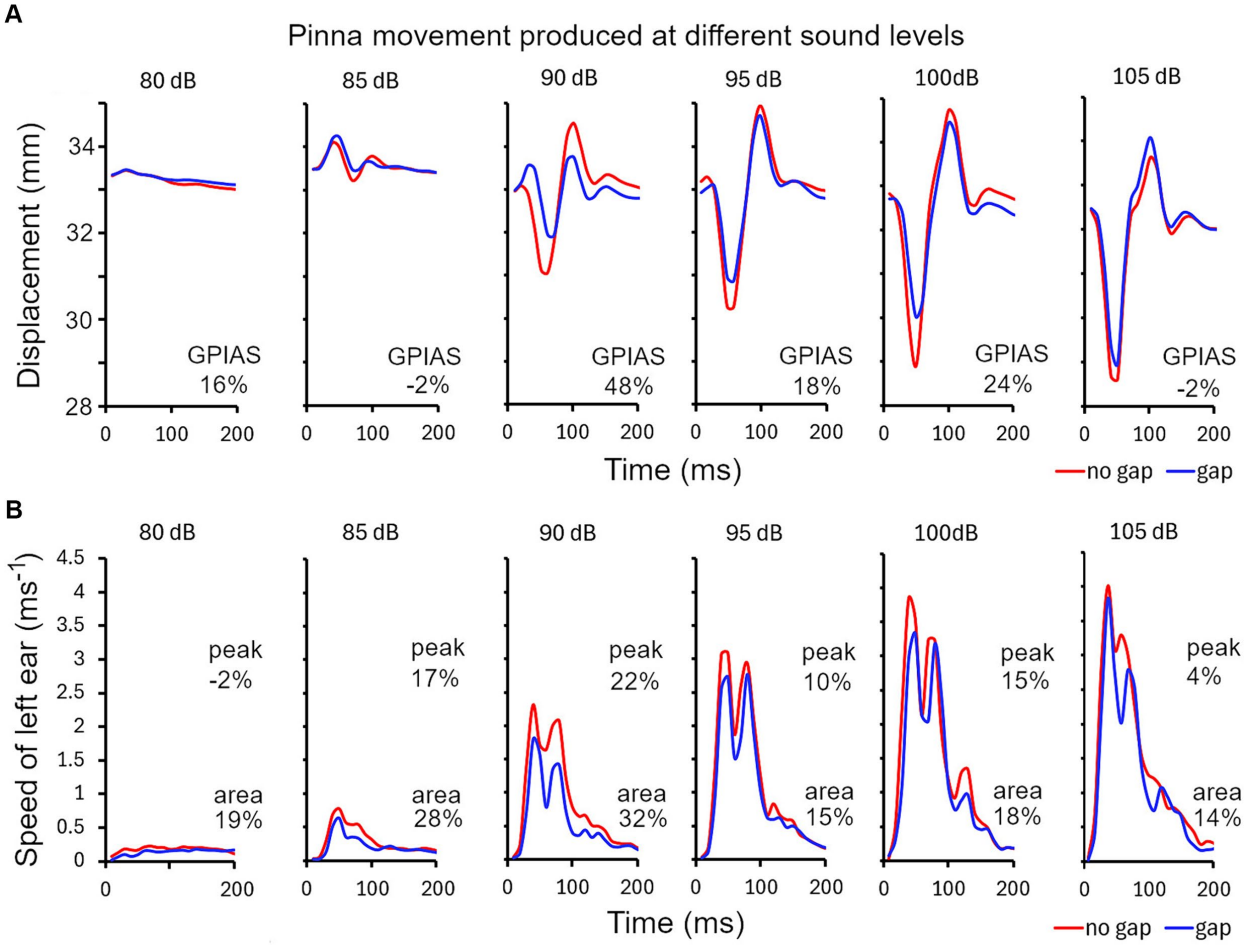
**(A)** Series of ear twitches measured as the distance between markers on the two ears, produced by startle pulses of different sound levels in 5 dB steps in ascending order, from 80 to 105 dB SPL for guinea pig 7DDF7B5. The pulses were superimposed on a narrow band (4–6 kHz) continuous background noise. Gaps were inserted at random immediately before half of the pulses. GPIAS is measured from the distance between the negative trough and positive peak for the gap and no-gap conditions and is expressed as a percentage, as shown for each panel. **(B)** An alternative way for calculating GPIAS is provided by measuring the speed (change in position of a single marker on one ear over the 10 ms sampling intervals). By comparing the peak speed or the area under the graph for the gap and no-gap conditions, it is possible to make a different estimate of percentage reduction produced by the gap (GPIAS) as indicated by the pairs of numbers in each panel.

There are various methods for analysing the pinna reflex as indicated above and we compared three of them in the guinea pig in one representative trial comprising 10 repeats of each of the gap and no-gap stimuli: (1) startle displacement measured as the difference between the maximum and minimum distance between the ears during the startle (Figure 4A); (2) peak startle speed, measured from the first differential of the position of a single ear (Figure 4B); (3) total movement, measured as the area under the curve of startle speed over the 200 ms time window (Figure 4B). During the startle response, the pinnae make a circular movement, in three dimensions, and when the ears are moving in parallel to each other the displacement change drops to 0 even though the speed is still relatively high. When displacement is measured between two ear markers there are at least three components: an initial positive wave (ears moving away from each other) followed by a negative wave (ears moving towards each other) and then another positive wave. The amplitudes of these waves vary depending on the startle intensity and are modified by the presence of a gap but also vary between animals. In the example shown in Figure 4A there was a relatively large initial positive wave followed by smaller negative and positive waves with an 85 dB SPL startle stimulus, whereas at 90 dB SPL and above the initial positive wave was smaller and was dominated by the much bigger negative and second positive waves. Both the negative and second positive waves were usually reduced in size by the presence of a gap before the startle pulse but sometimes only the negative wave was reduced. The degree of GPIAS varies depending on the sound level, and for this guinea pig was strongest between 90 and 100 dB SPL, with the reduction caused by the gap between −2 and 48% over the range of sound levels tested.

The peak startle speed of a single ear can be measured by plotting the first differential of position of a single ear as a function of time (Figure 4B). There were typically two main peaks in the speed curve and both of these were reduced by the presence of a preceding gap but only the changes in the largest peak were measured. The strongest GPIAS was present at the levels of 85–100 dB SPL with the peak analysis method and the reduction caused by the gap varied from −2 to 22% (upper numbers in Figure 4B). When the same data was analysed, by integrating the area under the graph for each sound level, changes in all the peaks were included to give a value for total movement and more consistent values for GPIAS were obtained that varied between 14 and 32% (lower numbers in Figure 4B). All three methods agree that the strongest GPIAS is produced with startle pulses at a sound level of 90 dB SPL for this guinea pig.

**Figure 5:**
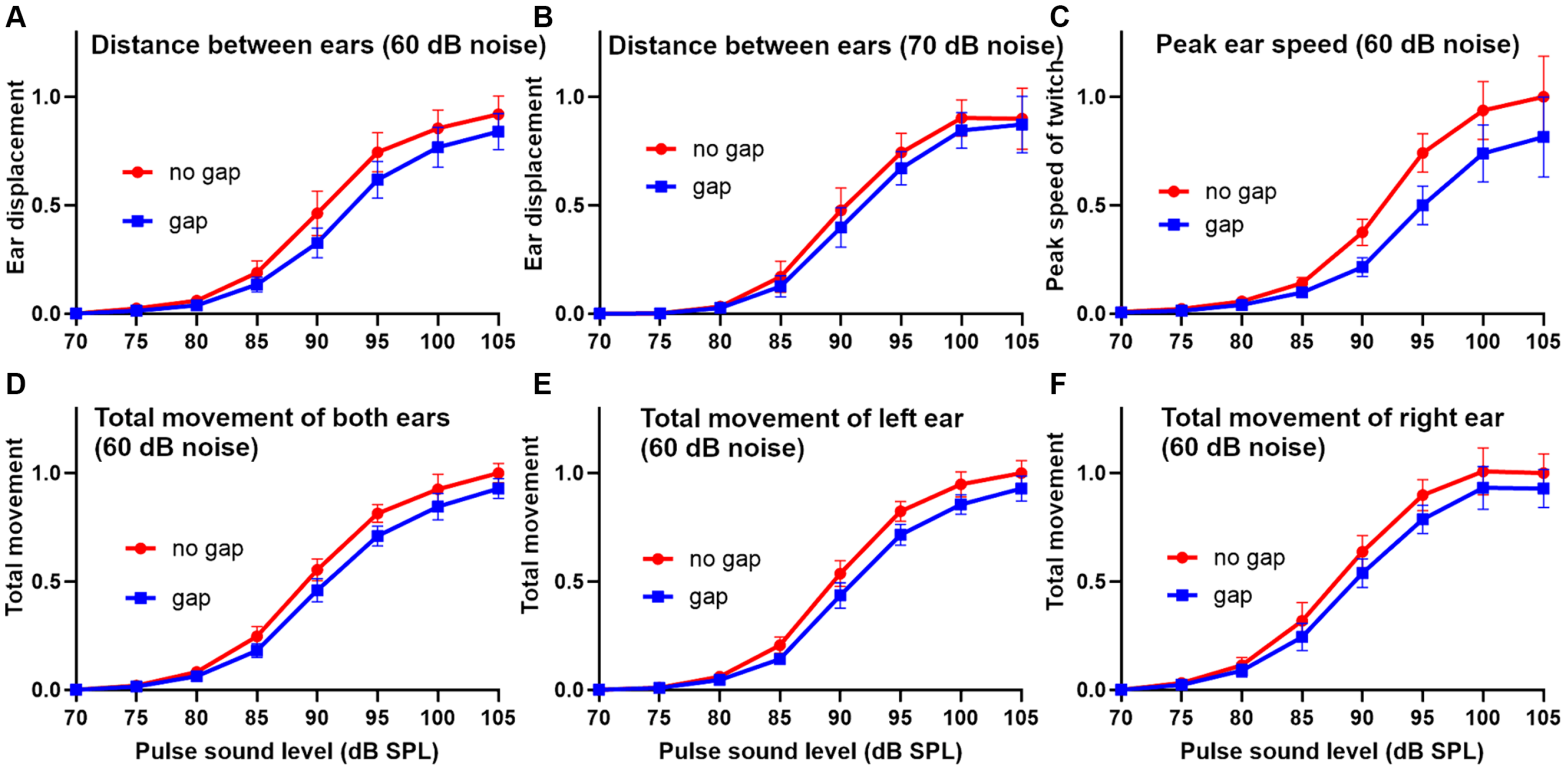
Sigmoid-shaped plots showing the relationship between pinna reflex characteristics and the sound level of the startle pulse in a group of 10 guinea pigs for the gap and no gap conditions. Error bars show SEM. The reflexes were analyzed in three different ways after normalizing the results. In the first method the displacement (change in distance between the two ear markers) was plotted using background noise (4–6 kHz) at 60 dB SPL **(A)** or 70 dB SPL **(B)**. In the second method the peak speed (first differential of location) for each individual ear marker was combined for the two ears and plotted for a 60 dB SPL noise background **(C)**. In the third method the total reflex movement (integrated area under the curve of reflex speed over a period of 250 ms) is shown as the mean of the left and right ears **(D)**, left ear alone **(E)** or right ear alone **(F)**.

To obtain a better overall idea of the differences between the different analysis methods, we applied them to data from 10 guinea pigs and combined them to study the growth curves of response amplitude for increasing sound levels of the stimulus pulse after normalising the results to the maximum value (Figure 5). The means for the gap (blue) and no gap (red) conditions for the different pulse levels were compared by paired t-test. The sigmoid growth curves of pinna reflex displacement show the difference between the gap and no gap results at a background noise level of 60 dB SPL (Figure 5A) and 70 dB SPL (Figure 5B). The curves with a background of 60 dB SPL showed stronger GPIAS values at most pulse levels than were obtained with a background of 70 dB SPL. Overall, the difference between the two curves was more strongly significant across the population at 60 dB SPL (*p* = 0.005) than at 70 dB SPL (*p* = 0.014). When the peak reflex speed was measured by combining the results from both ears with a background of 60 dB SPL, there was a greater separation between the two curves (Figure 5C) but there was also a greater variability, and the separation probability was less significant (*p* = 0.017). The differences between the startle values for peak reflex speed were also greater than those for total reflex movement obtained by measuring the area under the curve of reflex speed (Figures 5D–F). Despite this, lower variability meant that the difference between gap and no-gap was significantly different when the values for the two ears were combined at a 60 dB SPL background (*p* = 0.004) or the left ear measured alone (*p* = 0.006), or the right ear alone (*p* = 0.002). In conclusion, all three methods for analysing the response show significant values for GPIAS and are equally valid ways of demonstrating it.

### 3.4 Displacement of abdominal, back, tail and ear markers in mice

**Figure 6:**
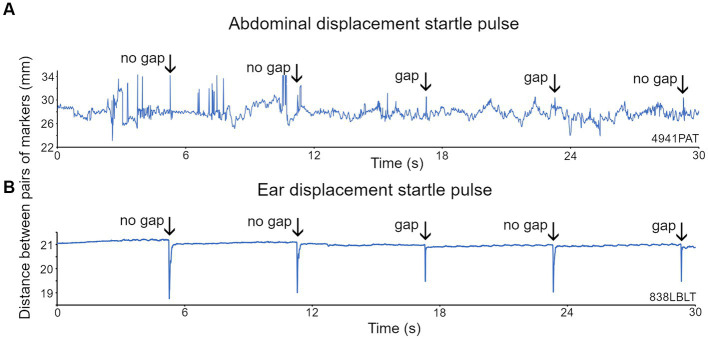
Recordings of the displacement of two markers placed on either side of the abdomen and on each ear of the mouse. **(A)** For the abdominal markers, startle pulses at 100 dB SPL with a background of broadband noise (BBN) at 60 dB SPL were presented with or without a preceding gap. Abdominal twitches in response to the startle pulses are indicated by the arrows. **(B)** Ear twitches (marked by arrows) in response to startle pulses of 105 dB SPL on a BBN background of 70 dB SPL are more prominent than the abdominal twitches, because although the ear movements are smaller, there is much less background movement.

Reflective markers were placed on both sides of the abdomen but in mice one was often either removed or hidden by the body orientation. However, a clear GPIAS signal could typically be obtained from a single abdominal marker (Figure 6A). The startle response was identified by placing a response window over a period of 150 ms starting from the stimulus onset. We attempted to place markers on the pinna, as in guinea pigs, but unlike the more placid guinea pigs, mice rapidly removed markers from the pinna, and therefore it was usually impractical to use pinna markers in mice. In two mice that retained an ear marker it was possible to observe GPIAS similar to that shown above for guinea pigs (Figure 6B).

**Figure 7:**
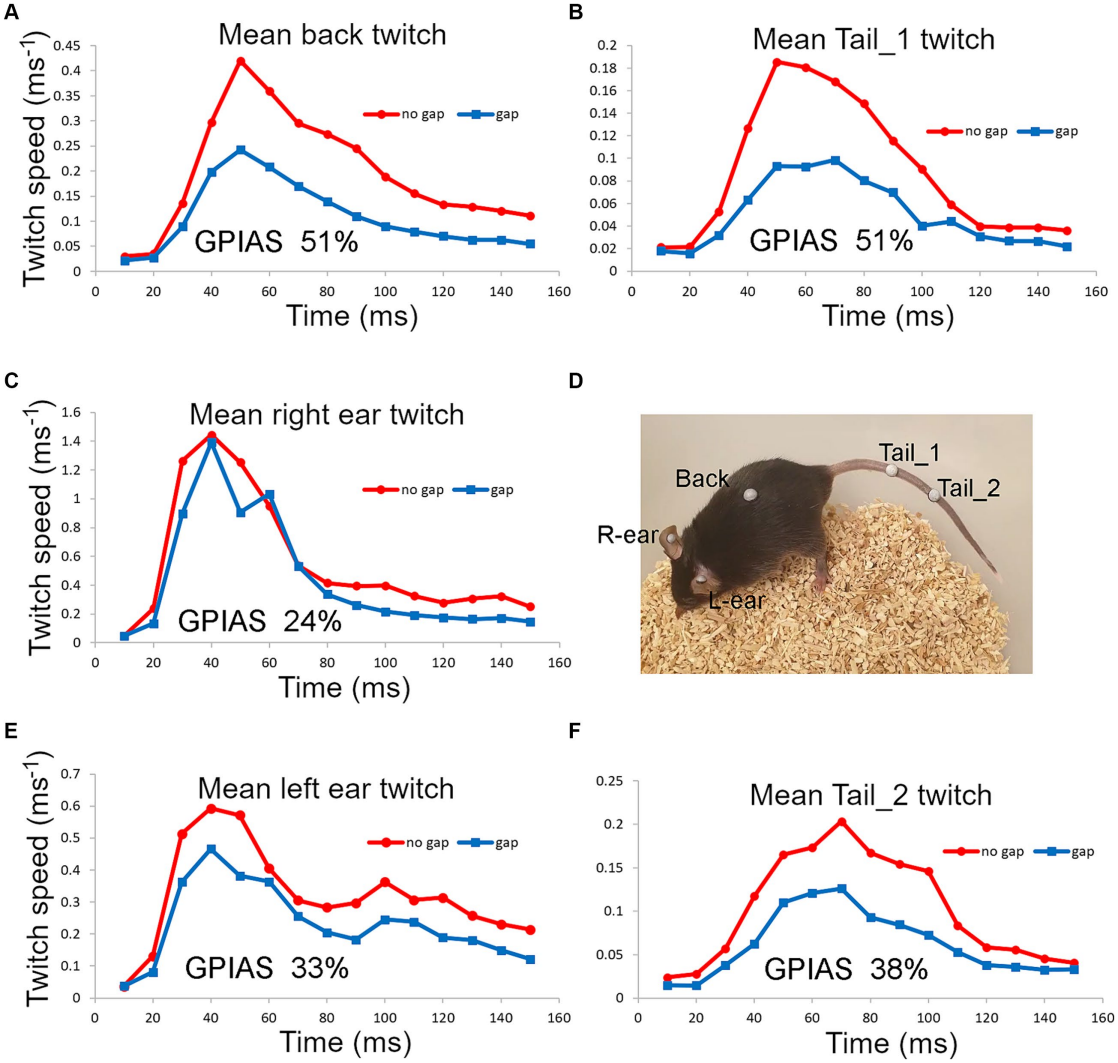
Startle movements were simultaneously compared in one mouse (838LBLT) by placing reflective markers, at five different locations **(D)**, involving the ears **(C,E)**, back **(A)** and tail **(B,F)**. The twitch speed was plotted for each marker and summed over a 150 ms window for 50 presentations of the gap condition interspersed with 50 presentations of the no gap condition, using BBN background at 70 dB SPL and a pulse strength of 105 dB SPL. This gave a value for the total movement at each location and allowed the amount of GPIAS to be compared.

**Figure 8:**
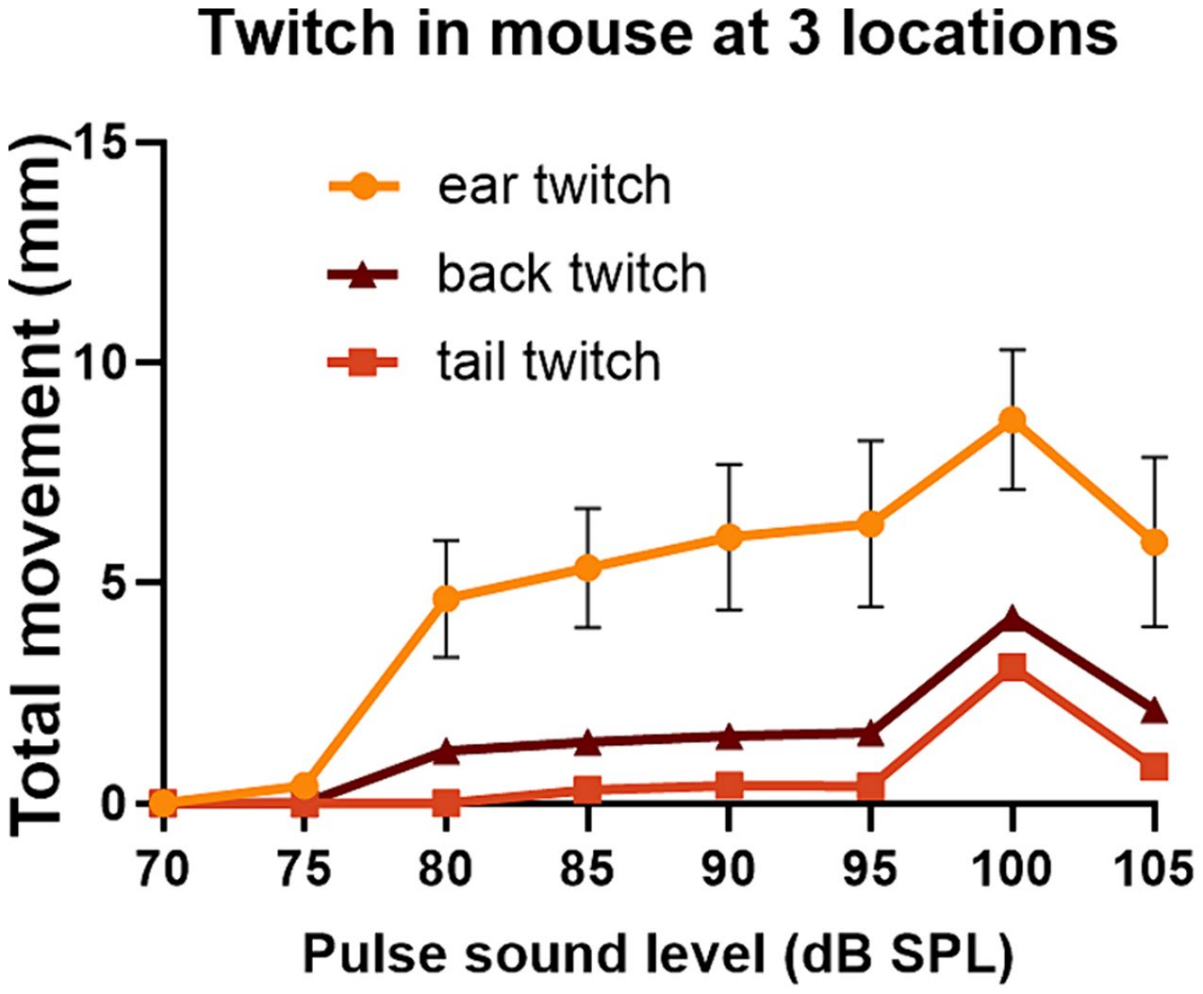
The same mouse (838LBLT) also allowed us to compare the growth curves for the twitch strength (total movement) at different locations when startle pulses were presented at different levels. The error bars show SEM and were too small to plot for the back and tail. The growth curves had a similar shape for the ears, back and tail and similar thresholds for startle movement at around 75 dB SPL. The ear twitches had much greater amplitude but also showed much more variability than the twitches at the back and tail (2 repeats at each sound level with mean values given for the two ears and two tail markers).

We also placed two markers on the tail and one on the back as these seemed to be the locations that were best tolerated by the mice. For one mouse that tolerated ear, back and tail markers simultaneously, we were able to directly compare GPIAS recorded from three locations. The results are shown in Figure 7 where the total movement of the twitch was measured for each marker in gap and no gap conditions. For this mouse (Figure 7D), GPIAS was stronger when recorded from the back (Figure 7A) and tail markers (Figures 7B,F) than by the ear markers (Figures 7C,E). Startle sequences were presented to this mouse at a range of startle pulse sound levels from 75 to 105 dB SPL (Figure 8). The shape of the growth curve was similar for each location of marker and the thresholds were also similar at about 75 dB SPL. The results are consistent with the suggestion that the back and tail twitches are equally valid to the pinna reflex for measuring the acoustic startle and GPIAS.

### 3.5 Effect of sodium salicylate on the strength of GPIAS in mice

A variety of salicylate doses have been used in mice to induce tinnitus and the optimal dose may vary with age or strain. We used a range of doses from 200 to 300 mg/kg as doses above this caused distress, in contrast to guinea pigs in which 350 mg/kg was used routinely (Berger et al., 2013, 2017; Wallace et al., 2021).

**Figure 9:**
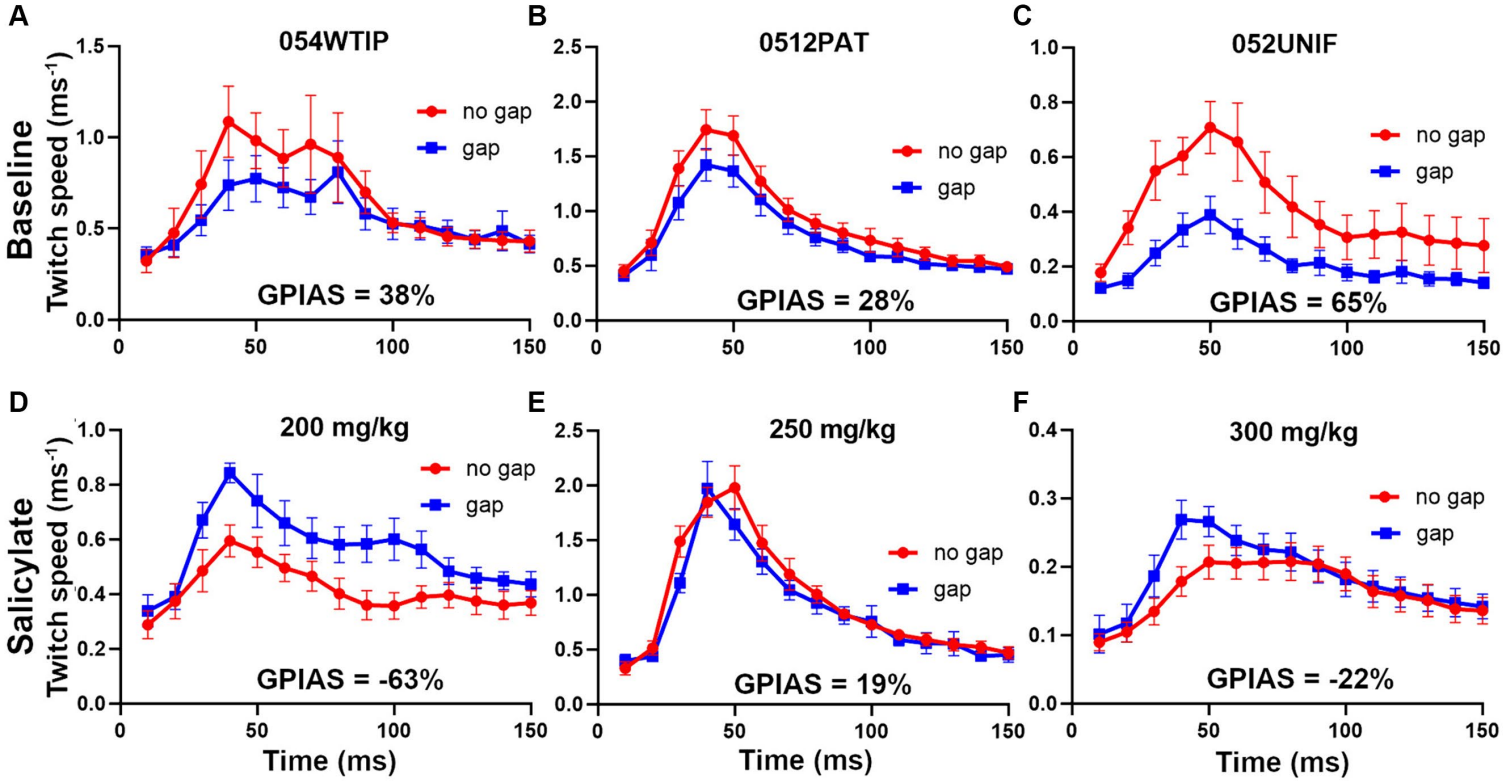
Examples of the changes in GPIAS produced by three different doses of sodium salicylate (s.c.). The upper panel for each mouse **(A–C)** shows the baseline GPIAS using a background of BBN at 70 dB SPL and startle pulses at 105 dB SPL based on a combination of any back and tail markers still present. The error bars show the SEM. The plots show the change in marker speed over a period of 150 ms during the response to the gap and no gap conditions. In each case there is a significant reduction in the twitch response when the startle pulse is preceded by a gap in the background noise. The strength of the twitch was measured as the total movement (area under curve). The lower panels show the responses for the same animals after injection of sodium salicylate **(D)** 200 mg/kg, **(E)** 250 mg/kg and **(F)** 300 mg/kg, when the gap condition gives a similar or bigger response compared to the no gap condition.

Prior to the injections of salicylate, all mice were tested for GPIAS and only mice showing reliable GPIAS (determined by paired t-tests on the mean twitch waveform for the gap and no-gap responses for each trial) were subjected to salicylate injections. To demonstrate GPIAS we placed three reflective markers on the mouse: one on the neck or back and two on the tail, prior to a motion-tracking session in the sound booth. The mice often tried to remove the markers and they were sometimes replaced between individual trials. However, in some cases markers came off during a trial and complete data was only obtained for one or two markers. The data from different markers were combined for each trial and were all given equal weighting so that a mean was obtained from all the markers available. Examples of the changes in GPIAS obtained following injection of salicylate at different doses of between 200 and 300 mg/kg are shown in Figure 9. Prior to the injection of salicylate there was evidence of significant GPIAS ranging from 28 to 65%. The significance of the GPIAS before and after salicylate injections was determined by a repeated-measures, two-way ANOVA. The differences between the means for the gap and no gap conditions at baseline, for the three mice in Figure 9, were as follows: Figure 9A, *F* (1, 240) = 4.2 and *p* = 0.04; Figure 9B, *F* (1, 390) = 13.3 and *p* = 0.0003; Figure 9C, *F* (1, 300) = 53.3 and *p* < 0.0001. Two hours after the injection of salicylate there was a significant reduction in the amount of GPIAS, with values between 19% to-63% (pre-pulse facilitation). The differences between the means for the gap and no gap conditions at baseline, for the three mice, 2 h after the injection of salicylate, were as follows: Figure 9D, facilitation *F* (1, 150) = 44.4 and *p* < 0.0001; Figure 9E, inhibition *F* (1, 330) = 3.8 and *p* = 0.052; Figure 9F, facilitation *F* (1, 360) = 6.05 and *p* = 0.01. The presence of gap-induced facilitation after salicylate was not typical as most mice just showed a reduction in GPIAS, but these results illustrate the variability between mice.

**Figure 10:**
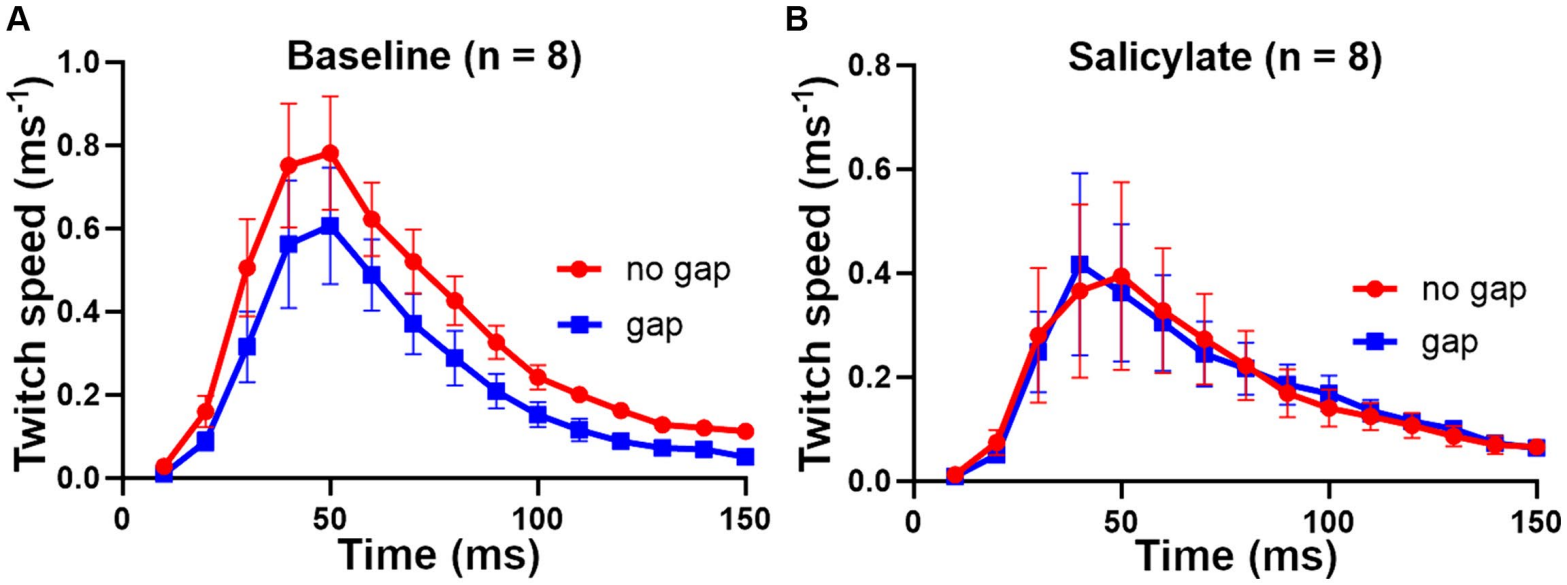
Combined results for the 8 mice that received sodium salicylate at doses of 200 to 300 mg/kg based on a combination of back and tail markers. **(A)** At baseline the 8 mice showed highly significant GPIAS, with the mean difference between gap and no gap being 32% [*F* (1, 210) = 17.2 and *p* < 0.0001]. **(B)** After salicylate treatment, there was no evidence of GPIAS, consistent with the presence of tinnitus being induced by salicylate, with mean difference 0.7% [*F* (1, 210) = 0.002 and *p* = 0.96]. Error bars show SEM.

There was no clear effect of increasing salicylate dose in the range 200–300 mg/kg, so we combined the results from the 8 mice from the three dosage groups to show a clear effect of the salicylate in abolishing the GPIAS (Figure 10). The percentage difference between the gap and no gap conditions at baseline, for the eight mice in Figure 10A, was 32% (*p* < 0.0001), showing a highly significant value for gap detection before exposure to salicylate. By contrast the percentage difference between the combined means for the gap and no gap conditions after salicylate, for the same eight mice (Figure 10B), was 0.7%, *p* = 0.96, showing that exposure to salicylate had abolished GPIAS.

## 4 Discussion

### 4.1 Demonstration of the acoustic startle reflex in mice and guinea pigs

Our first aim was to develop a reliable method for measuring GPIAS in the mouse, that would avoid using the whole-body startle. We had previously used an expensive Vicon system to measure GPIAS in guinea pigs by placing reflective markers on their pinnae and measuring the Preyer reflex, a component of the acoustic startle (Preyer, 1882). We have now replaced the Vicon system with a much less expensive, but just as accurate, OptiTrack motion tracking system. Initially, we tried restraining mice and placing markers on their pinnae. However, the restraint was stressful and almost as soon as they were released, most mice removed the markers by grooming movements with their forepaws. Consequently, we considered alternative methods of measuring the acoustic startle reflex that would avoid both any restraint and the sensitive head region. An alternative way for measuring the pinna reflex in the guinea pig is to use spots of green paint that are tracked by a conventional video camera (Martel et al., 2018; Martel and Shore, 2020). However, this only tracks in two dimensions and has a refresh rate of 50 Hz which is unsuitable for following rapid twitches in the mouse. Faster cameras, with sampling at 150 Hz, have been used to study changes in mouse facial expression, without the use of markers, but this required fixation of the head (Clayton et al., 2024). Thus, we sought a less sensitive location on the body where markers would have a better chance of being left in place by a freely moving mouse.

The acoustic startle reflex automatically protects mammals from an external threat by rapidly activating many muscle groups, throughout the entire body, mainly via the large motoneurons of the caudal pontine reticular formation (Gómez-Nieto et al., 2014). In primates this involves stiffening the limbs, body wall and dorsal neck (Wilkins et al., 1986; Yeomans and Frankland, 1995), blinking the eye by activating muscles around and within the orbit (Rogenmoser et al., 2022; Smith et al., 2024), flattening the pinna against the head (Hackley, 2015) and inhibiting the masseter muscle (Meier-Ewert et al., 1974; Deriu et al., 2007). In the mouse it also involves muscle extension of the limbs as well as a recoil of the head and ears, changes in facial expression, hunching of the back and twitching of the tail as demonstrated by high-speed photography (Pantoni et al., 2020; Clayton et al., 2024). Initially we placed markers on the head, back, thorax and abdomen of guinea pigs. We confirmed that all the markers recorded rapid, short-latency twitches in response to a startle pulse (Figure 2). The body twitches had a similar threshold to the pinna reflex and although the movements were smaller (Figure 3), they appeared suitable for study in mice as the variability was also less. Unfortunately, the mice usually removed markers on the head, pinna and neck within 20 s of being put in place. Only the back and tail markers were less frequently removed by grooming as they were on areas of less sensitive skin. At each of these positions the threshold for the twitch was similar to the pinna at 75–80 dB SPL (Figure 8) and they all showed similar growth curves, with an increase in the speed of the twitch as the startle pulse level increased.

When the guinea pig Preyer reflex was studied in detail it was found to be composed of different components. At low sound levels (85 dB SPL) there was an initial movement that increased the separation between the two ears but at higher sound levels the ears initially moved towards each other and then apart so that there was a late overshoot in separation (Figure 4A). When the speed of a single ear was plotted for these twitches, there were two or three peaks (Figure 4B). The rapid, dynamic nature of the movements indicates that they are the result of active muscle contractions rather than mechanical oscillations. In the case of the rat there are five separate muscles that act on the pinna (Friauf and Herbert, 1985) and similar muscles appear to act in the guinea pig where there are separate branches of the facial nerve that innervate the anterior and posterior auricular muscles (Uemura-Sumi et al., 1986). In the rat, the neurons in the facial nucleus that innervate the auricular muscles receive a direct and indirect input from the cochlear root nucleus that is thought to form the basis of the pinna response to an acoustically produced startle (Horta-Júnior et al., 2008). The ear displacement and peak speed of the pinna reflex can show considerable variation between trials and this is partly due to the timing and the force of the various pinna muscles acting against each other with the different muscles being under independent control. It could also be partly due to separate neural pathways contributing to the startle reflex with different latencies (Wilson et al., 2019). Various limb muscles are also activated in antagonistic groups during the startle response (Wilkins et al., 1986) and this may be true of muscles controlling the head, back and torso as the twitches produced at these locations always had at least two components (Figure 2).

When measuring the pinna reflex in guinea pigs previously (Berger et al., 2013), we used a measure of the maximum change in the distance between the two ears (displacement), but in this study we also measured the maximum speed of a single pinna movement as well as the total movement of an individual marker measured by integrating the area under the movement graph (Figure 4B). In the guinea pig, we found that the peak speed and total movement of a pinna gave equally consistent and useful measures of GPIAS as pinna displacement, and so we used these methods of analysis on single markers in the mouse as well. The differences between the gap and no-gap intensity growth curves for ear displacement (Figures 5A,B) were smaller than we expected based on our previous guinea pig work. This may have been because the current work was with the Dunkin-Hartley strain, whereas previously we had used a tricolour strain and previously we discarded animals that did not produce a strong GPIAS response in initial testing whereas in this study we retained all the animals to study the growth curves.

### 4.2 Identification of tinnitus using the GPIAS method with salicylate

Originally tinnitus was thought to “fill in” the gap in background noise that acted to inhibit the subsequent response to the acoustic startle (Turner et al., 2006) but that was found not to be the case. Groups of humans and animals with tinnitus were still able to detect gaps in background noise almost as well as control groups matched for hearing loss (Galazyuk and Hébert, 2015). Instead, the GPIAS test seems able to identify changes in sensory motor gating that are specifically related to the gap-induced inhibition and involve a pathway to the auditory cortex (Bowen et al., 2003) rather than a pathway through the lateral globus pallidus that is involved in pre-pulse inhibition (Moreno-Paublete et al., 2017). It seems that the plastic changes in the ascending auditory pathway and the caudal pontine reticular formation associated with tinnitus (Chen et al., 2017), directly affect the pathways controlling GPIAS. Thus, GPIAS in guinea pigs, as measured by a behavioural assay, is also associated with gap induced inhibition of the cortical evoked potential produced by a startling stimulus and both are altered by the presence of tinnitus (Berger et al., 2018).

GPIAS is thought to be a reliable way of measuring tinnitus in guinea pigs but its use in mice has been more controversial. Recent work using a model of noise overexposure to induce tinnitus in mice had led to the suggestion that GPIAS was not a reliable method for detecting tinnitus when using the whole-body startle (WBS), at least in CBA mice (Fabrizio-Stover et al., 2022). Previously, the detailed study by Longenecker et al. (2018) showed that when GPIAS is measured in mice with the WBS there is considerable variability in the response. They showed that the strength of GPIAS is altered by a wide range of factors including habituation, inter-trial interval, inter-stimulus interval, circadian rhythm, sex differences and sensory adaptation. Another major factor is mouse strain (Yu et al., 2016) because C57BL/6 mice show much stronger GPIAS than the CBA mice which were used in the noise overexposure studies cited above. A further confounding factor is the fact that noise exposure may produce tinnitus in less than half of the treated animals. We aimed to overcome some of these problems of response reliability by avoiding the WBS and using a more reliable method of inducing tinnitus.

One of the simplest and most reliable ways of inducing tinnitus is by administering a high dose of salicylate (Cazals, 2000; Stolzberg et al., 2012). Salicylate is an effective anti-inflammatory agent and analgesic in rats and presumably other rodents (Jablonski and Howden, 2002). Thus, our mice showed little irritation following the salicylate injection. The doses of salicylate that have previously been used to induce tinnitus in mice include 200 mg/kg (Yanagawa et al., 2017), 250 mg/kg (Gröschel et al., 2016) and 300 mg/kg (Guitton, 2009; Yu et al., 2016). Doses higher than this have been used in the past, but they were associated with distress and eventually death in some animals (Thurston et al., 1970; Fiume, 2003). The LD_50_ for salicylate in mice does not seem to have been determined accurately, but it is advisable in our experience to use doses of less than 350 mg/kg to induce tinnitus in order to avoid signs of distress. Salicylate has a complex set of actions by acting on the cochlea to increase thresholds and reduce overall output, particularly at high frequencies (Wallace et al., 2021), but also by acting directly on the brain to increase gain control in the auditory pathway and increase spontaneous activity in non-auditory structures such as the hippocampus and reticular formation (Salvi et al., 2021). Salicylate apparently enters the brain and can suppress inhibition, particularly of GABA in the cortex where it leads to enhanced acoustic responses (Sun et al., 2009) and altered tonotopic maps (Yanagawa et al., 2017). When we tested for GPIAS before and after the injection of salicylate in our mice, the GPIAS present in the baseline condition was always reduced by salicylate, but by very variable amounts. We only found a significant reduction in the GPIAS level, reliably, when results from three or more mice were averaged. This confirmed that the acoustic startle reflex measured by twitches on the back and tail is a suitable method for identifying the presence of tinnitus in groups of mice. The GPIAS was not always completely abolished by salicylate even in groups of three mice, but when results from a larger group of mice (10) were averaged, we found complete abolition of GPIAS. Thus, a significant reduction in GPIAS was a correlate of salicylate induced tinnitus, but the absence of a significant reduction in GPIAS could not be taken as conclusive proof that tinnitus was not present in an individual. In order for the GPIAS method to be used to detect tinnitus in individual mice, it would need to be improved. This could be done by optimising along the lines suggested by Longenecker et al. (2018), possibly by reducing the inter-stimulus interval and randomising the inter-trial interval for the stimulation as well as taking into account the best strain, the best time of day and factors related to sex.

The other development required to validate motion tracking based GPIAS, as a reliable indicator of tinnitus in mice and other rodents, is to develop a parallel method for use in humans, where tinnitus can be confirmed using a questionnaire. Humans do not have tails and their pinnae can only make small curling movements of 2–3 mm (Hackley, 2015), but people are prepared to have small reflective markers placed on their face. In humans, the acoustic startle reflex involves changes in muscles controlled by the facial nerve (Wilkins et al., 1986; Smith et al., 2024) and these should be detectable by motion tracking. The OptiTrack system has already been used to measure small movements of the mandible (Furtado et al., 2013) and skin deformation produced by spinal movements (Beaudette et al., 2017). This suggests that reflective markers placed on the human face or neck could also be used to measure GPIAS using remote cameras in the same way as guinea pigs and mice and could allow translational studies.

## 5 Conclusion

Recording from markers placed on the back and tail of mice proved to be remarkably sensitive at detecting the small, rapid twitches associated with the acoustic startle response. These twitches showed little sign of habituation within a single trial and had less variability than pinna and abdominal movements, and so were routinely used to measure the measure GPIAS. Injection of salicylate, at concentrations thought to induce tinnitus in mice, was found to completely suppress GPIAS when results were grouped together. This work validates GPIAS as a useful tool in quantifying tinnitus in mice, opening up possible future use of transgenic mouse models to study the molecular basis of tinnitus.

## Data availability statement

The raw data supporting the conclusions of this article will be made available by the authors, without undue reservation.

## Ethics statement

The animal study was approved by Animal Welfare and Ethical Review Body at the University of Nottingham, UK. The study was conducted in accordance with the local legislation and institutional requirements.

## Author contributions

MW: Conceptualization, Data curation, Formal analysis, Investigation, Methodology, Project administration, Visualization, Writing – original draft, Writing – review & editing. JB: Formal analysis, Methodology, Software, Writing – review & editing. AH: Conceptualization, Methodology, Writing – review & editing. CS: Methodology, Project administration, Writing – review & editing. MA: Data curation, Project administration, Resources, Supervision, Writing – review & editing. AP: Funding acquisition, Project administration, Resources, Supervision, Validation, Writing – review & editing. PM: Conceptualization, Funding acquisition, Project administration, Supervision, Writing – review & editing.
